# Supplementary material for: Real-world outcomes for a complete nationwide cohort of more than 3200 teriflunomide-treated multiple sclerosis patients in The Danish Multiple Sclerosis Registry
Source: PLoS One. 2021 May 18;16(5):e0250820. doi: 10.1371/journal.pone.0250820 (PMC8130956; doi:10.1371/journal.pone.0250820)
Supplement: S1 Table — (DOCX) [file pone.0250820.s001.docx]

| **S1 Table.** **Temporal changes in the use of teriflunomide in Denmark** | | | | | | | | | | |
| --- | --- | --- | --- | --- | --- | --- | --- | --- | --- | --- |
|  | Total | 2013 | 2014 | 2015 | 2016 | 2017 | 2018 | 2019 | p-value^1^ | Missing |
|  | *(n=3239)* | *(n=31)* | *(n=711)* | *(n=821)* | *(n=603)* | *(n=497)* | *(n=388)* | *(n=188)* |  |  |
| Sex, *n (%)* |  |  |  |  |  |  |  |  | 0.0175 | 0 |
| Male | 1120 (34.6) | 13 (41.9) | 284 (39.9) | 270 (32.9) | 197 (32.7) | 159 (32.0) | 125 (32.2) | 72 (38.3) |  |  |
| Female | 2119 (65.4) | 18 (58.1) | 427 (60.1) | 551 (67.1) | 406 (67.3) | 338 (68.0) | 263 (67.8) | 116 (61.7) |  |  |
| Age at disease onset, *median (IQR), mean (SD)** | 34.2 (26.6 ; 41.4)  34.5 (10.1) | 33.2 (26.5 ; 42.9)  34.8 (10.9) | 33.3 (25.9 ; 40.1)  33.5 (9.6) | 34.7 (27.3 ; 41.4)  34.7 (9.8) | 33.8 (26.2 ; 41.7)  34.4 (10.5) | 34.6 (26.7 ; 42.6)  35.2 (10.4) | 34.4 (27.3 ; 42.1)  35.2 (10.6) | 34.7 (27.1 ; 41.4)  34.8 (9.9) | 0.0963 | 11 |
| Age at TFL start, *median (IQR), mean (SD)** | 42.9 (35.2 ; 50.1)  42.6 (10.7) | 43.2 (36.7 ; 54.7)  44.8 (12.2) | 43.5 (37.0 ; 50.2)  43.4 (10.1) | 43.1 (36.2 ; 49.5)  42.8 (10.2) | 41.8 (33.7 ; 50.5)  42.0 (11.2) | 42.7 (33.9 ; 50.3)  42.3 (11.1) | 42.7 (33.7 ; 50.3)  42.1 (11.4) | 43.0 (34.0 ; 48.8)  41.8 (10.6) | 0.1494 | 0 |
| Time since disease onset, *median (IQR), mean (SD)** | 5 (1 ; 12)  7.55 (8.13) | 8 (2 ; 17)  9.48 (8.21) | 7 (3 ; 14)  9.36 (8.03) | 5 (1 ; 12)  7.64 (7.93) | 4 (1 ; 11)  7.06 (8.22) | 3 (1 ; 10)  6.58 (7.92) | 2 (1 ; 10)  6.40 (8.35) | 2 (1 ; 11)  6.53 (8.15) | <0.0001 | 11 |
| Time since disease onset, *n (%)* |  |  |  |  |  |  |  |  | <0.0001 | 11 |
| 0 years | 511 (15.8) | 3 (9.7) | 51 (7.2) | 118 (14.4) | 109 (18.1) | 103 (20.8) | 87 (22.5) | 40 (21.7) |  |  |
| >0 years | 2717 (84.2) | 28 (90.3) | 659 (92.8) | 702 (85.6) | 493 (81.9) | 391 (79.2) | 300 (77.5) | 144 (78.3) |  |  |
| Time since diagnosis, *median (IQR), mean (SD)** | 1 (0 ; 7)  4.41 (6.34) | 3 (1 ; 9)  5.48 (6.52) | 4 (1 ; 10)  6.25 (6.67) | 1 (0 ; 7)  4.38 (6.15) | 0 (0 ; 6)  3.84 (6.17) | 0 (0 ; 5)  3.35 (5.82) | 0 (0 ; 5)  3.61 (6.14) | 0 (0 ; 5)  3.65 (6.67) | <0.0001 | 0 |
| Time since diagnosis, *n (%)* |  |  |  |  |  |  |  |  | <0.0001 | 0 |
| 0 years | 1436 (44.3) | 7 (22.6) | 151 (21.2) | 345 (42.0) | 311 (51.6) | 290 (58.4) | 227 (58.5) | 105 (55.9) |  |  |
| >0 years | 1803 (55.7) | 24 (77.4) | 560 (78.8) | 476 (58.0) | 292 (48.4) | 207 (41.6) | 161 (41.5) | 83 (44.1) |  |  |
| Diagnosis, *n (%)* |  |  |  |  |  |  |  |  | <0.0001 | 0 |
| CIS | 215 (6.6) | 0 (0.0) | 13 (1.8) | 52 (6.3) | 38 (6.3) | 60 (12.1) | 46 (11.9) | 6 (3.2) |  |  |
| MS | 3024 (93.4) | 31 (100.0) | 698 (98.2) | 769 (93.7) | 565 (93.7) | 437 (87.9) | 342 (88.1) | 182 (96.8) |  |  |
| Previous treatment, *n (%)* |  |  |  |  |  |  |  |  | <0.0001 | 0 |
| No | 1824 (56.3) | 6 (19.4) | 203 (28.6) | 420 (51.2) | 399 (66.2) | 367 (73.8) | 286 (73.7) | 143 (76.1) |  |  |
| Yes | 1415 (43.7) | 25 (80.6) | 508 (71.4) | 401 (48.8) | 204 (33.8) | 130 (26.2) | 102 (26.3) | 45 (23.9) |  |  |
| Previous treatment, *n (%)* |  |  |  |  |  |  |  |  | 0.8351 | 0 |
| Moderate efficacy DMT | 1360 (96.1) | 25 (100.0) | 488 (96.1) | 386 (96.3) | 194 (95.1) | 127 (97.7) | 97 (95.1) | 43 (95.6) |  |  |
| High efficacy DMT | 55 (3.9) | 0 (0.0) | 20 (3.9) | 15 (3.7) | 10 (4.9) | 3 (2.3) | 5 (4.9) | 2 (4.4) |  |  |
| Number of DMTs before TFL treatment start, *n (%)* |  |  |  |  |  |  |  |  | <0.0001 | 0 |
| None | 1824 (56.3) | 6 (19.4) | 203 (28.6) | 420 (51.2) | 399 (66.2) | 367 (73.8) | 286 (73.7) | 143 (76.1) |  |  |
| 1 | 793 (24.5) | 17 (54.8) | 305 (42.9) | 238 (29.0) | 109 (18.1) | 64 (12.9) | 41 (10.6) | 19 (10.1) |  |  |
| 2 | 389 (12.0) | 8 (25.8) | 127 (17.9) | 100 (12.2) | 61 (10.1) | 38 (7.7) | 41 (10.6) | 14 (7.5) |  |  |
| 3 | 149 (4.6) | 0 (0.0) | 45 (6.3) | 42 (5.1) | 20 (3.3) | 21 (4.2) | 12 (3.1) | 9 (4.8) |  |  |
| 4 | 53 (1.6) | 0 (0.0) | 23 (3.2) | 12 (1.5) | 10 (1.7) | 5 (1.0) | 3 (0.8) | 0 (0.0) |  |  |
| >4 | 31 (1.0) | 0 (0.0) | 8 (1.1) | 9 (1.1) | 4 (0.7) | 2 (0.4) | 5 (1.3) | 3 (1.6) |  |  |
| EDSS at TFL start, *median (IQR), mean (SD)* | 2.0 (1.0 ; 3.0)  2.13 (1.46) | 2.0 (1.5 ; 3.0)  2.24 (1.51) | 2.0 (1.5 ; 3.0)  2.32 (1.57) | 2.0 (1.5 ; 3.0)  2.16 (1.39) | 2.0 (1.0 ; 3.0)  2.13 (1.53) | 2.0 (1.0 ; 2.5)  1.95 (1.36) | 2.0 (1.0 ; 2.5)  1.90 (1.39) | 2.0 (1.0 ; 2.5)  2.04 (1.32) | 0.0014 | 412 |
| ARR at TFL start *median (IQR), mean (SD)* | 0 (0 ; 1)  0.53 (0.62) | 0 (0 ; 0)  0.19 (0.40) | 0 (0 ; 1)  0.29 (0.51) | 0 (0 ; 1)  0.46 (0.63) | 1 (0 ; 1)  0.69 (0.66) | 1 (0 ; 1)  0.66 (0.65) | 1 (0 ; 1)  0.65 (0.59) | 1 (0 ; 1)  0.65 (0.54) | <0.0001 | 0 |
| Number of relapses in year prior to TFL start, *n (%)* |  |  |  |  |  |  |  |  | <0.0001 | 0 |
| 0 | 1736 (53.6) | 25 (80.7) | 524 (73.7) | 499 (60.8) | 247 (41.0) | 211 (42.5) | 159 (41.0) | 71 (37.8) |  |  |
| ≥1 | 1503 (46.4) | 6 (19.3) | 187 (26.3) | 322 (39.2) | 356 (59.0) | 286 (57.5) | 229 (59.0) | 117 (62.2) |  |  |

ARR: annualized relapse rate, CIS: clinically isolated syndrome, DMT: disease-modifying therapy, EDSS: Expanded Disability Status Scale, IQR: interquartile range, MS: multiple sclerosis, n: number, SD: standard deviation, TLF: teriflunomide. *in years. ^1^p-value of a test comparing the patients starting TFL in the various years; a chi-squared test is used for categorical characteristics and a non-parametric Kruskal-Wallis test is used for continuously values characteristics.
