# Supplementary material for: Real-world outcomes for a complete nationwide cohort of more than 3200 teriflunomide-treated multiple sclerosis patients in The Danish Multiple Sclerosis Registry
Source: PLoS One. 2021 May 18;16(5):e0250820. doi: 10.1371/journal.pone.0250820 (PMC8130956; doi:10.1371/journal.pone.0250820)
Supplement: S2 Table — (DOCX) [file pone.0250820.s002.docx]

| **S2 Table. One-year follow-up and risk factors for adverse event and treatment escalation** | | | | | | | | | | | | | |
| --- | --- | --- | --- | --- | --- | --- | --- | --- | --- | --- | --- | --- | --- |
|  |  |  |  |  |  | Unadjusted | | | | Adjusted^3^ | | | |
|  | One year after start of TFL treatment | | | | | Adverse events |  | Escalation |  | Adverse events |  | Escalation |  |
|  | Still under treatment | Stopped because of adverse events | Escalated | Other | p-value^1^ | HR^2^ (95%CI) | p-value | HR^2^ (95%CI) | p-value | HR^2^ (95%CI) | p-value | HR^2^ (95%CI) | p-value |
|  | *(n=2182)* | *(n=469)* | *(n=234)* | *(n=116)* |  |  |  |  |  |  |  |  |  |
| Sex |  |  |  |  | <0.0001 |  |  |  |  |  |  |  |  |
| Male | 795 (77.6) | 104 (10.2) | 91 (8.9) | 34 (3.3) |  | 0.57  (0.48-0.68) | <0.0001 | 1.32  (1.13-1.55) | 0.0004 | 0.61  (0.50-0.74) | <0.0001 | 1.24  (1.05-1.46) | 0.0128 |
| Female | 1387 (70.2) | 365 (18.5) | 143 (7.2) | 82 (4.2) |  | ref |  | ref |  | ref |  | ref |  |
| Age at disease onset, *median (IQR), mean (SD)** | 34.5 (26.7 ; 41.7)  34.7 (10.3) | 34.4 (28.3 ; 41.5)  34.9 (9.2) | 31.8 (23.8 ; 39.0)  32.0 (9.6) | 31.4 (25.2 ; 41.8)  33.6 (10.9) | 0.0004 | 1.04  (0.97-1.11) | 0.3225 | 0.75  (0.69-0.81) | <0.0001 | 1.09  (1.00-1.19) | 0.0634 | 0.64  (0.59-0.70) | <0.0001 |
| Age at TFL start, *median (IQR), mean (SD)** | 43.1 (35.6 ; 50.4)  42.9 (10.6) | 44.5 (38.0 ; 51.7)  44.5 (10.4) | 36.9 (29.3 ; 44.7)  37.1 (10.2) | 41.4 (31.6 ; 49.0)  41.2 (11.6) | <0.0001 | 1.18  (1.10-1.26) | <0.0001 | 0.62  (0.57-0.66) | <0.0001 | - | - | - | - |
| Disease duration at TFL start, *median (IQR), mean (SD)** | 5 (1 ; 12)  7.69 (8.08) | 7 (2 ; 14)  9.11 (8.58) | 1 (0 ; 6)  4.66 (7.24) | 4 (1 ; 11)  7.09 (7.70) | <0.0001 | 1.23  (1.13-1.33) | <0.0001 | 0.63  (0.55-0.71) | <0.0001 | 1.11  (0.98-1.26) | 0.1116 | 0.61  (0.52-0.71) | <0.0001 |
| Disease duration (years) at TFL start |  |  |  |  | <0.0001 |  |  |  |  |  |  |  |  |
| <1 year | 302 (67.3) | 56 (12.5) | 75 (16.7) | 16 (3.6) |  | ref |  | ref |  | ref |  | ref |  |
| ≥1 years | 1875 (73.6) | 412 (16.2) | 159 (6.3) | 100 (3.9) |  | 1.42  (1.11-1.81) | 0.0048 | 0.48  (0.40-0.58) | <0.0001 | 1.09  (0.81-1.46) | 0.5634 | 0.66  (0.53-0.84) | 0.0005 |
| Time since diagnosis (10 years) | 1 (0 ; 7)  4.49 (6.32) | 2 (0 ; 10)  5.49 (6.62) | 0 (0 ; 1)  2.06 (4.84) | 1 (0 ; 7)  4.55 (6.51) | <0.0001 | 1.30  (1.17-1.44) | <0.0001 | 0.54  (0.46-0.64) | <0.0001 | 0.91  (0.72-1.15) | 0.4147 | 0.76  (0.59-0.98) | 0.0322 |
| Time since diagnosis |  |  |  |  | <0.0001 |  |  |  |  |  |  |  |  |
| 0 year | 937 (72.1) | 161 (12.4) | 154 (11.9) | 48 (3.7) |  | ref |  | ref |  | ref |  | ref |  |
| >0 years | 1245 (73.2) | 308 (18.1) | 80 (4.7) | 68 (4.0) |  | 1.53  (1.30-1.81) | <0.0001 | 0.53  (0.46-0.62) | <0.0001 | 1.09  (0.81-1.47) | 0.5664 | 0.78  (0.60-1.01) | 0.0560 |
| Diagnosis |  |  |  |  | 0.2935 |  |  |  |  |  |  |  |  |
| CIS | 156 (77.6) | 24 (11.9) | 12 (6.0) | 9 (4.5) |  | 0.72  (0.51-1.03) | 0.0706 | 0.55  (0.36-0.82) | 0.0039 | 0.86  (0.57-1.28) | 0.4559 | 0.41  (0.26-0.64) | 0.0001 |
| MS | 2026 (72.4) | 445 (15.9) | 222 (7.9) | 107 (3.8) |  | ref |  | ref |  | ref |  | ref |  |
| Treatment naive |  |  |  |  | <0.0001 |  |  |  |  |  |  |  |  |
| Yes | 1195 (72.5) | 211 (12.8) | 178 (10.8) | 64 (3.9) |  | ref |  | ref |  | ref |  | ref |  |
| No | 987 (73.0) | 258 (19.1) | 56 (4.1) | 52 (3.8) |  | 1.51  1.30-1.77) | <0.0001 | 0.57  (0.49-0.67) | <0.0001 | - | - | - | - |
| Previous treatment |  |  |  |  | 0.0374 |  |  |  |  |  |  |  |  |
| Moderate efficacy DMT | 957 (73.6) | 241 (18.5) | 52 (4.0) | 50 (3.9) |  | ref |  | ref |  | ref |  | ref |  |
| High efficacy DMT | 30 (56.6) | 17 (32.1) | 4 (7.6) | 2 (3.8) |  | 1.80  (1.15-2.83) | 0.0109 | 0.87  (0.43-1.77) | 0.7011 | 1.35  (0.78-2.35) | 0.2865 | 0.73  (0.33-1.62) | 0.4309 |
| Reason for discontinuation of previous treatment |  |  |  |  | 0.4270 |  | 0.0208 |  | 0.072 |  | 0.3123 |  | 0.4703 |
| Adverse events | 644 (72.0) | 185 (20.7) | 30 (3.4) | 36 (4.0) |  | ref |  | ref |  | ref |  | ref |  |
| Disease breakthrough | 32 (76.2) | 7 (16.7) | 2 (4.8) | 1 (2.4) |  | 0.75  (0.38-1.49) | 0.4184 | 1.30  (0.64-2.61) | 0.4687 | 0.92  (0.46-1.88) | 0.8260 | 1.08  (0.48-2.44) | 0.8602 |
| Pregnancy | 33 (78.6) | 5 (11.9) | 2 (4.8) | 2 (4.8) |  | 0.39  (0.16-0.97) | 0.0427 | 1.75  (0.92-3.31) | 0.0862 | 0.52  (0.21-1.30) | 0.1610 | 1.14  (0.59-2.22) | 0.6911 |
| Other | 278 (74.3) | 61 (16.3) | 22 (5.9) | 13 (3.5) |  | 0.73  (0.57-0.94) | 0.0148 | 1.37  (1.04-1.80) | 0.0262 | 0.83  (0.64-1.09) | 0.1772 | 1.27  (0.95-1.71) | 0.1132 |
| Number of DMTs before TFL treatment start |  |  |  |  | <0.0001 |  | <0.0001 |  | <0.0001 |  | 0.0006 |  | 0.0626 |
| None | 1195 (72.5) | 211 (12.8) | 178 (10.8) | 64 (3.9) |  | ref |  | ref |  | ref |  | ref |  |
| 1 | 569 (74.3) | 132 (17.2) | 38 (5.0) | 27 (3.5) |  | 1.37  (1.14-1.65) | 0.0009 | 0.57  (0.47-0.69) | <0.0001 | 1.38  (1.09-1.75) | 0.0070 | 0.79  (0.62-0.99) | 0.0391 |
| 2 | 277 (75.1) | 69 (18.7) | 7 (1.9) | 16 (4.3) |  | 1.45  (1.15-1.83) | 0.0016 | 0.52  (0.40-0.68) | <0.0001 | 1.39  (1.05-1.85) | 0.0230 | 0.72  (0.53-0.99) | 0.0407 |
| 3 | 94 (68.1) | 31 (22.5) | 8 (5.8) | 5 (3.6) |  | 1.87  (1.36-2.58) | 0.0001 | 0.83  (0.59-1.17) | 0.2916 | 1.78  (1.23-2.58) | 0.0022 | 1.12  (0.75-1.68) | 0.5842 |
| 4 | 33 (62.3) | 17 (32.1) | 1 (1.9) | 2 (3.8) |  | 2.80  (1.81-4.36) | <0.0001 | 0.37  (0.17-0.82) | 0.0143 | 2.52  (1.55-4.09) | 0.0002 | 0.49  (0.22-1.10) | 0.0832 |
| >4 | 14 (51.9) | 9 (33.3) | 2 (7.4) | 2 (7.4) |  | 2.59  (1.41-4.76) | 0.0023 | 0.38  (0.12-1.22) | 0.1053 | 2.07  (1.08-4.00) | 0.0293 | 0.54  (0.17-1.75) | 0.3033 |
| EDSS at treatment start with any DMT | 2.0 (1.0 ; 2.5)  1.96 (1.10) | 2.0 (1.0 ; 3.0)  2.16 (1.27) | 2.0 (1.3 ; 2.5) ; 2.04 (1.22) | 2.0 (1.0 ; 3.0)  2.19 (1.49) | 0.0033 | 1.12  (1.05-1.19) | 0.0004 | 0.97  (0.91-1.03) | 0.2573 | 1.04  (0.96-1.14) | 0.3296 | 1.01  (0.92-1.11) | 0.8584 |
| EDSS at TFL start | 2.0 (1.0 ; 2.5)  2.07 (1.45) | 2.0 (1.5 ; 3.0)  2.43 (1.51) | 2.0 (1.0 ; 2.5)  2.14 (1.38) | 2.0 (1.5 ; 3.0)  2.42 (1.68) | <0.0001 | 1.13  (1.07-1.19) | <0.0001 | 0.96  (0.91-1.01) | 0.1426 | 1.06  (1.01-1.13) | 0.0343 | 1.10  (1.04-1.17) | 0.0013 |
| ARR at TFL start | 0 (0 ; 1)  0.48 (0.61) | 0 (0 ; 1)  0.50 (0.64) | 1 (0 ; 1)  0.85 (0.69) | 0 (0 ; 1)  0.53 (0.63) | <0.0001 | 0.89  (0.78-1.02) | 0.0907 | 1.62  (1.46-1.81) | <0.0001 | 1.09  (0.93-1.27) | 0.2865 | 1.30  (1.12-1.51) | 0.0007 |
| Number of relapses in year prior to TFL start, n (%) |  |  |  |  | <0.0001 |  |  |  |  |  |  |  |  |
| 0 | 1243 (75.6) | 268 (16.3) | 71 (4.3) | 62 (3.8) |  | ref |  | ref |  | ref |  | ref |  |
| ≥1 | 939 (69.2) | 201 (14.8) | 163 (12.0) | 54 (4.0) |  | 0.83  (0.71-0.97) | 0.0177 | 1.84  (1.58-2.14) | <0.0001 | 1.12  (0.91-1.39) | 0.2829 | 1.24  (1.01-1.52) | 0.0427 |

ARR: annualized relapse rate, CIS: clinically isolated syndrome, DMT: disease-modifying therapy, EDSS: Expanded Disability Status Scale, HR: hazard ratio, IQR: interquartile range, MS: multiple sclerosis, n: number, SD: standard deviation, TLF: teriflunomide. *10-year. ^1^P-value of a test comparing treatment status one year after start of TFL treatment; a chi-squared test is used for categorical characteristics and a non-parametric Kruskal-Wallis test is used for continuously values characteristics. ^2^Ratio of the subdistribution hazards for the corresponding ending event. ^3^Adjusted for sex, age at disease onset, disease duration at TFL treatment start, number of DMT before TFL treatment start, EDSS at TFL treatment start, ARR the year before TFL treatment start.
