# Supplementary material for: Real-world outcomes for a complete nationwide cohort of more than 3200 teriflunomide-treated multiple sclerosis patients in The Danish Multiple Sclerosis Registry
Source: PLoS One. 2021 May 18;16(5):e0250820. doi: 10.1371/journal.pone.0250820 (PMC8130956; doi:10.1371/journal.pone.0250820)
Supplement: Attachment — Submitted filename: Response to the reviewers.docx [file pone.0250820.s003.docx]

Journal Requirements:

When submitting your revision, we need you to address these additional requirements.

1. Please ensure that your manuscript meets PLOS ONE's style requirements, including those for file naming. The PLOS ONE style templates can be found at [https://journals.plos.org/plosone/s/file?id=wjVg/PLOSOne_formatting_sample_main_body.pdf](https://eur01.safelinks.protection.outlook.com/?url=https%3A%2F%2Fjournals.plos.org%2Fplosone%2Fs%2Ffile%3Fid%3DwjVg%2FPLOSOne_formatting_sample_main_body.pdf&data=04%7C01%7CSvend.Geertsen%40sanofi.com%7Ce02e1328a1364c49e04808d8fa0e5380%7Caca3c8d6aa714e1aa10e03572fc58c0b%7C0%7C0%7C637534287862157925%7CUnknown%7CTWFpbGZsb3d8eyJWIjoiMC4wLjAwMDAiLCJQIjoiV2luMzIiLCJBTiI6Ik1haWwiLCJXVCI6Mn0%3D%7C1000&sdata=lGjDWkdKsn%2BZXnOE7NCt7rIbUh6bAzJ5xkdQpnHU8WE%3D&reserved=0) and [https://journals.plos.org/plosone/s/file?id=ba62/PLOSOne_formatting_sample_title_authors_affiliations.pdf](about:blank)

>>Response: We have carefully reviewed our manuscript and made sure it meets PLOS ONE’s style requirements.

2. Thank you for stating the following in the Financial Disclosure section:

'YES. The study was supported by Sanofi: medical writing and the fee of professional
statistician. But Sanofi had no role in study design or data collection.'

We note that one or more of the authors are employed by a commercial company: Sanofi.

1. Please provide an amended Funding Statement declaring this commercial affiliation, as well as a statement regarding the Role of Funders in your study. If the funding organization did not play a role in the study design, data collection and analysis, decision to publish, or preparation of the manuscript and only provided financial support in the form of authors' salaries and/or research materials, please review your statements relating to the author contributions, and ensure you have specifically and accurately indicated the role(s) that these authors had in your study. You can update author roles in the Author Contributions section of the online submission form.

Please also include the following statement within your amended Funding Statement.

“The funder provided support in the form of salaries for authors [insert relevant initials], but did not have any additional role in the study design, data collection and analysis, decision to publish, or preparation of the manuscript. The specific roles of these authors are articulated in the ‘author contributions’ section.”

If your commercial affiliation did play a role in your study, please state and explain this role within your updated Funding Statement.

>>Response: We have updated the Funding Statement accordingly:

“This study was funded by Sanofi. The funder provided support for medical writing and fee for a professional statistician, but the funder did not have any additional role in the study design, data collection and analysis or decision to publish. JvW and SSG are employees of Sanofi. The specific roles of these authors are articulated in the ‘author contributions’ section.”

2. Please also provide an updated Competing Interests Statement declaring this commercial affiliation along with any other relevant declarations relating to employment, consultancy, patents, products in development, or marketed products, etc.

Within your Competing Interests Statement, please confirm that this commercial affiliation does not alter your adherence to all PLOS ONE policies on sharing data and materials by including the following statement: "This does not alter our adherence to  PLOS ONE policies on sharing data and materials.” (as detailed online in our guide for authors [http://journals.plos.org/plosone/s/competing-interests](about:blank)) . If this adherence statement is not accurate and  there are restrictions on sharing of data and/or materials, please state these.

>>Response: We have updated the Competing Interests Statement accordingly:

“This study was funded by Sanofi. VP: has received support for scientific meetings from Merck and Sanofi Genzyme and honoraria for lecturing from Alexion. MDB: None. VS: None. PVR: has served on scientific advisory board for Biogen, Sanofi, Roche, Novartis, Merck, and Alexion, has received honoraria for lecturing from Biogen, Merck, Novartis, Roche, has received support for congress participation from Biogen, Genzyme, Roche, Merck, Novartis. ZI: has served on scientific advisory boards, received support for congress participation, received speaker honoraria, or received research support for his laboratory from Biogen, Merck, Roche, Sanofi Genzyme. MK: None. CH: has served on scientific advisory board for Biogen, Sanofi, Roche, Novartis, has received honoraria for lecturing from Biogen, Merck, Novartis, Sanofi, Genzyme, has received support for congress participation from Biogen and Roche. ZM: None. HR: None. TS: has served on scientific advisory boards, received support for congress participation, received speaker honoraria and received research support from Biogen and Novartis, and received support for congress participation by Roche. AW: has served on scientific advisory board for Merck, Biogen and Roche, has received honoraria for lecturing and publications from Sanofi Genzyme, Merck, Roche and has received support for congress participation from Biogen, Genzyme, Teva, Merck and Roche. JvW and SSG are employees of Sanofi. SB: has received support for congress participation from Biogen and Roche. FS: has served on scientific advisory boards, been on the steering committees of clinical trials, served as a consultant, received support for congress participation, received speaker honoraria, or received research support for his laboratory from Biogen, EMD Serono, Merck, Novartis, Roche, Sanofi Genzyme and Teva. MM: has served on scientific advisory board for Biogen, Sanofi, Roche, Novartis, Merck, Abbvie, Alexion has received honoraria for lecturing from Biogen, Merck, Novartis, Sanofi, Genzyme, has received research support and support for congress participation from Biogen, Genzyme, Roche, Merck, Novartis. The specific roles of these authors are articulated in the ‘author contributions’ section. With reference to PLOS ONE policies on sharing data and materials, we confirm that this does not alter our adherence to PLOS ONE policies on sharing data and materials.”

Please include both an updated Funding Statement and Competing Interests Statement in your cover letter. We will change the online submission form on your behalf.

Please know it is PLOS ONE policy for corresponding authors to declare, on behalf of all authors, all potential competing interests for the purposes of transparency. PLOS defines a competing interest as anything that interferes with, or could reasonably be perceived as interfering with, the full and objective presentation, peer review, editorial decision-making, or publication of research or non-research articles submitted to one of the journals. Competing interests can be financial or non-financial, professional, or personal. Competing interests can arise in relationship to an organization or another person. Please follow this link to our website for more details on competing interests: [http://journals.plos.org/plosone/s/competing-interests](about:blank)

Please review your reference list to ensure that it is complete and correct. If you have cited papers that have been retracted, please include the rationale for doing so in the manuscript text, or remove these references and replace them with relevant current references. Any changes to the reference list should be mentioned in the rebuttal letter that accompanies your revised manuscript. If you need to cite a retracted article, indicate the article’s retracted status in the References list and also include a citation and full reference for the retraction notice.

>>Response: We have included the updated Funding Statement and Competing Interests Statement in the Cover letter. We have also reviewed our reference list to ensure that it is complete and correct.

Reviewers' comments:

Reviewer's Responses to Questions

**Comments to the Author**

3. Have the authors made all data underlying the findings in their manuscript fully available?

The [PLOS Data policy](https://eur01.safelinks.protection.outlook.com/?url=http%3A%2F%2Fwww.plosone.org%2Fstatic%2Fpolicies.action%23sharing&data=04%7C01%7CSvend.Geertsen%40sanofi.com%7Ce02e1328a1364c49e04808d8fa0e5380%7Caca3c8d6aa714e1aa10e03572fc58c0b%7C0%7C0%7C637534287862157925%7CUnknown%7CTWFpbGZsb3d8eyJWIjoiMC4wLjAwMDAiLCJQIjoiV2luMzIiLCJBTiI6Ik1haWwiLCJXVCI6Mn0%3D%7C1000&sdata=er%2FJcQwhi2qstkdj39sTkL%2FE8%2B9fhEZqYP%2BeBC7M3WM%3D&reserved=0) requires authors to make all data underlying the findings described in their manuscript fully available without restriction, with rare exception (please refer to the Data Availability Statement in the manuscript PDF file). The data should be provided as part of the manuscript or its supporting information, or deposited to a public repository. For example, in addition to summary statistics, the data points behind means, medians and variance measures should be available. If there are restrictions on publicly sharing data—e.g. participant privacy or use of data from a third party—those must be specified.

Reviewer #1: No

>>Response: We have added the following statement on p. 8, l. 166: “In Denmark, registry data are kept at secured research servers Data sharing is not permitted according to Danish legislation. These data are, however, available on request by researchers meeting the criteria for access to confidential information..”

5. Review Comments to the Author

Reviewer #1: This article is an important addition to the literature and well-written. I only have two minor comments to note:

- Is a citation available to support the categorization of MS drugs into moderate and high efficacy?

>>Response: This is the categorization that is used in the official treatment guidelines in Denmark. We have added a reference: “Sorensen P, Kopp T, Joensen H, Olsson A, Sellebjerg F, Magyari M. Age and sex as determinants of treatment decisions in patients with relapsing-remitting MS. Mult Scler Relat Disord. 2021;50:102813.”

- Page 9, Line 167: I believe this is the first use of the acronym for annualized relapse rate in the text, but the acronym hasn't define yet. Also, a description of this measure and the calculation is not available in the Methods section.

>>Response: Thank you for noting this. We have now defined ARR at first use and added a description of this measure in the methods section on p. 6, l. 114: “...annualized relapse rate (ARR) at teriflunomide treatment start that counts the number of relapses in the year prior to teriflunomide treatment start. If time since disease onset is 1 year or less, the relapse count is forced to be at least 1.”
